# Increased Out-of-Hospital Cardiac Arrest Risk in Cancer: Evidence From 800,000 Patients and 3.7 Million Controls

**DOI:** 10.1016/j.jacadv.2026.103293

**Published:** 2026-10-05

**Authors:** Hanna L. Hägglund, Agneta Månsson Broberg, Martin Jonsson, Sandra Eloranta, Elham Hedayati, Christel Hedman, Therese Djärv

**Affiliations:** a Department of Medicine Solna, Karolinska Institutet, Stockholm, Sweden; b Karolinska University Hospital, Stockholm, Sweden; c Department of Medicine Huddinge, Karolinska Institutet, Stockholm, Sweden; d Department of Cardiology, Karolinska University Hospital, Stockholm, Sweden; e Center for Resuscitation Science, Department of Clinical Science and Education, Karolinska Institutet Södersjukhuset, Stockholm, Sweden; f Division of Clinical Epidemiology, Department of Medicine Solna, Karolinska Institutet, Stockholm, Sweden; g Department of Oncology-Pathology, Karolinska Institute, Stockholm, Sweden; h South General Hospital, Department of Oncology, Stockholm, Sweden; i Department of Molecular Medicine and Surgery, Karolinska Institutet, Stockholm, Sweden; j R&D Department, Stockholms Sjukhem Foundation, Stockholm, Sweden; k Institute for Palliative Care, Region Skåne Department of Clinical Sciences Lund, Lund University, Lund, Sweden

**Keywords:** cardio-oncology, cardiovascular disease, population-based cohort study

## Abstract

**Background:**

Although cancer patients face an increased risk of cardiovascular disease, their risk of out-of-hospital cardiac arrest (OHCA) remains poorly characterized.

**Objectives:**

The objective of the study was to investigate the hazard of OHCA among patients with cancer and identify clinical factors associated with increased hazard compared with cancer-free controls.

**Methods:**

This population-based, matched cohort study included adults (≥18 years) diagnosed with cancer from 2010 to 2022, matched 1:5 on age, sex, and region, to cancer-free controls. Using 7 national registers, we assessed the hazard of OHCA for all cancers combined and across subgroups, including cancer site, metastasis status, and cardiovascular risk factors, using Cox proportional hazards models and model-based estimates of cumulative incidence.

**Results:**

A total of nearly 800,000 patients with cancer and 3,700,000 controls were included. Cancer was associated with a 55% higher hazard of OHCA (adjusted HR: 1.55; 95% CI: 1.50-1.60), than controls. The greatest excess hazards were observed in lung, gastrointestinal, and neurological cancers. The association was strongest within the first year after cancer diagnosis, but remained increased throughout follow-up. Lung and neurological cancers were associated with the greatest short-term hazards, approximately 6-fold higher than controls, whereas lung cancer and multiple myeloma showed the highest long-term excess hazards, approximately 2- to 3-fold higher.

**Conclusions:**

Patients with cancer had a higher hazard of OHCA than cancer-free controls. Cancer site, time since diagnosis, and metastasis status were key factors associated with the hazard of OHCA. These findings may help inform tailored cardiovascular risk assessment, preventive strategies, shared decision-making, and end-of-life planning in oncology care.

Over the past decades, improved cancer survival has increased recognition of related noncancer morbidity and mortality, particularly those of cardiovascular origin.^1^^,^^2^ Cancer survivors face a markedly elevated risk of cardiovascular disease (CVD) and cardiovascular mortality,^3^^,^^4^ driven by shared risk factors and cardiotoxic effects related to several oncological therapies.^5^^,^^6^ The European Society of Cardiology published its first cardio-oncology guidelines in 2022, in collaboration with the International Cardio-Oncology Society, to optimize cardiovascular care before, during, and after cancer therapy.^1^

Despite the elevated cardiovascular risk among cancer survivors, the association between cancer and out-of-hospital cardiac arrest (OHCA) remains poorly characterized. Today, OHCA is a major global health concern,^7^ and is defined within this study as the sudden cessation of cardiac function occurring outside a hospital setting and achieving chest compression or defibrillation.^8^^,^^9^ Prior studies have focused mainly on outcomes after OHCA among patients with cancer,^10^, ^11^, ^12^, ^13^ with inconsistent results, and we previously reported poorer short- and long-term overall survival after OHCA among cancer patients.^10^ Still, robust evidence on the risk and determinants of OHCA among cancer patients is limited.

Identifying cancer patients at high risk of OHCA is essential for prevention, tailored cardiovascular care, and shared decision-making and informed end-of-life care discussions. Therefore, we aimed to investigate the risk of OHCA among cancer patients compared with cancer-free controls, and to identify clinical factors associated with elevated risk. We hypothesized that cancer increases the risk of OHCA, with risk varying by cancer site, time since diagnosis, and baseline cardiovascular risk.

## Methods

### Study design and setting

This matched cohort study was based on multiple national registers. Sweden has a population of approximately 10 million, with about 70,000 malignant tumor cases diagnosed annually,^14^ and 6,000 OHCA cases each year. The health care system is decentralized and primarily funded through taxation, resulting in minimal out-of-pocket costs for patients.^15^^,^^16^ To promote equitable cancer care, Sweden has established national care programs that provide recommendations across the cancer care continuum.^17^ These national care programs are widely implemented and demonstrate high adherence among Swedish oncologists.^17^

### Data sources

For this study, we used 7 national, population-based registers (Supplemental Figure 1). The *Swedish Cancer Register* records primary tumors based on clinical and histopathological reports^14^ and is estimated to cover more than 95% of cancers in most regions.^18^ The *Register of the* T*otal* P*opulation* covers 100% of births and deaths, and 95% and 91% of immigrations and emigrations, respectively.^19^ The *Swedish Register of Cardiopulmonary Resuscitation*, a national quality register, includes all OHCA cases in which cardiopulmonary resuscitation is initiated by emergency medical services (EMS) personnel or bystanders, except for cases with definitive signs of death on EMS arrival. The register is estimated to have 90% coverage.^20^^,^^21^ The *National Patient Register* includes all inpatient care and specialized outpatient care, and has an estimated median positive predictive value of ∼84%, depending on diagnosis.^22^ The *National Prescribed Drug Register* comprehensively covers all prescribed drugs dispensed at pharmacies nationwide, but does not include most cancer treatments.^23^ The *Longitudinal Integrated Database for Health Insurance and Labour Market Studies* includes information on education and income. Education data are available for >98% of individuals, with an estimated accuracy of 85%.^24^ The *National Cause of Death Register* includes all deaths.^25^

### Study population

The study cohort consisted of adults (≥18 years) with a malignancy diagnosis registered in the *Swedish Cancer Register*, between January 1, 2010, and December 31, 2022. Tumors recorded as benign in the register were excluded from the study (Supplemental Figure 1). Five population controls per patient were sampled at random from the *Register of the Total Population*, matched by sex, age, region, and calendar period. Controls were required to be resident in Sweden and cancer-free on the matching date (ie, the date of cancer diagnosis for the corresponding patient), and to meet the specified matching criteria, including the same age as the patient on that date. Sampling was performed with replacement across risk sets, thus, a control individual could be sampled for multiple risk sets but could only be selected once for a given patient. Individual-level linkage between the national registers was performed using the unique Swedish personal identity number assigned to all Swedish residents.^26^ Linkage was performed by relevant authorities before the data were pseudonymized and made available for analysis.

### Exposure and outcome

The primary exposure was having been diagnosed with cancer, defined as any cancer diagnosis registered in the *Swedish Cancer Register*. In the primary analysis of all cancers combined, participants were included based on their first cancer diagnosis recorded during follow-up. Cancer site-specific subgroup analyses were subsequently performed (see Supplemental Table 1 for included subgroup definitions), in which participants with multiple cancer diagnosis were allowed to contribute to each relevant subgroup. The primary outcome was OHCA, registered in the *Swedish Register of Cardiopulmonary Resuscitation* during follow-up.

### Data collection and follow-up

The cohort was linked back to the *Swedish Cancer Register*, the *National Patient Register*, and the *Prescribed Drug Register* for identification of comorbidity; the *National Cause of Death Register* for dates of death; and *the Longitudinal Integrated Database for Health Insurance and Labour Market Studies –database* for identification of socioeconomic factors, using unique Swedish personal identity numbers.^26^ Finally, the cohort was linked to *the Swedish Register of Cardiopulmonary Resuscitation* for identification of the outcome of OHCA.

The cohort was followed from the date of inclusion (date of cancer diagnosis or matching date for controls) until an event (first recorded OHCA) or censoring, whichever happened first. Censoring occurred at death or at the end of follow-up (administrative censoring on December 31, 2022). Controls were also censored if diagnosed with cancer during follow-up.

### Statistical analysis

Baseline characteristics were presented as numbers and proportions for categorical variables and medians and IQRs for continuous variables. Standardized mean differences were used to assess baseline differences between groups. Unadjusted incidence rates of OHCA per 1,000 person-years with 95% CIs were estimated for cases and controls, respectively. In cases where the cancer diagnosis and OHCA occurred on the same day (n = 463), a 1-day follow-up period was added to prevent the exclusion of valid events. A sensitivity analysis excluding these cases was also performed. Adjusted HRs with 95% CIs, comparing patients with cancer and controls were estimated using Cox proportional hazard models. Robust (sandwich) SEs, clustered by individual ID, were applied to account for potential dependence in the data structure arising from controls being sampled with replacement, whereby controls could contribute to multiple risk sets. Models were adjusted for prespecified confounding factors (directed acyclic graph)^27^ (Supplemental Figure 2). These included adjustment for the matching covariates age at diagnosis or matching (using a natural cubic spline with 5 knots), and sex (man/woman, except for sex-specific cancers), whereas region was used only as a matching criteria to improve comparability with respect to potential geographical differences in health care access. Furthermore, adjustments were made for the unweighted Charlson Comorbidity Index^28^ (excluding cancer) at baseline, baseline CVD, as described subsequently, the calendar year of cancer diagnosis or matching, and socioeconomic factors. Baseline CVD was defined as a dichotomous variable (yes/no), based on the individual’s history of heart failure, ischemic heart disease (IHD), hypertension, or arrhythmia, or at least 1 dispensation of cardiovascular treatment (Supplemental Table 2). Baseline comorbidity (Charlson Comorbidity Index and CVD) was defined as a disease registered within 5 years before the date of inclusion. Socioeconomic factors included income and the highest level of education attained (0-9 years, 10-12 years, and ≥13 years). Income was defined as disposable household income per consumption unit, with household income adjusted for household composition using a standardized equivalence scale,^29^ categorized into quintiles. To account for potential income changes before cancer diagnosis,^30^ we used the mean income over 5 years before the date of inclusion. Missing values (Supplemental Table 3) were handled using a complete case analysis. Effect modification by baseline characteristics was investigated by including interaction terms between these covariates and the main exposure (cancer/control) to estimate stratum-specific HRs. The Wald test was used to formally assess the statistical significance of the interactions. To address nonproportional hazards, we also studied effect modification by follow-up time, by prespecified time intervals (≤6 months, >6 months to ≤1 year, >1 year to ≤2 years, and >2 years), that allowed HRs to vary across clinically interpretable follow-up periods. In exploratory analyses, separate Cox regression models were estimated for cancer groups defined according to shared etiological risk factors (Supplemental Table 4) and metastasis status (Supplemental Table 3), defined as tumor, node, metastasis (TNM) stage M1 or International Federation of Gynecology and Obstetrics (FIGO) stage IV in the *Swedish Cancer Register*. For classification into shared cardiovascular risk factor-related etiological groups,^31^ patients with cancers associated with more than 1 risk factor were included in each relevant group. The Cox proportional hazards models were used for the main analyses because our primary objective was to estimate the etiologic association between cancer and the hazard of OHCA. However, to estimate the absolute risk of OHCA while accounting for the competing risk of death, we also estimated cumulative incidence functions using Fine-Gray subdistribution hazards models. All analyses were performed using R software (the R Foundation for Statistical Computing; version 4.4.1). *P* values of <0.05 were regarded as statistically significant.

### Ethics

The study was approved by the Swedish Ethical Review Authority (Dnr 2023-07268-01) and was conducted in accordance with the Strengthening the Reporting of Observational Studies in Epidemiology Statement guidelines.^32^

## Results

### Baseline characteristics

We included 798,451 patients with a cancer diagnosis and 3,662,942 matched cancer-free controls (Supplemental Table 3). Among those who experienced OHCA, patients with cancer were younger, had a lower comorbidity burden, and had slightly more favorable socioeconomic factors than controls (Table 1). Among patients with cancer, those who experienced OHCA had a higher comorbidity burden, less favorable socioeconomic factors, and a higher prevalence of baseline CVD and cardiovascular treatment than those without OHCA. Among patients with cancer, the most common cancer sites were prostate, breast, and colorectal (Supplemental Table 5). The mean follow-up time was 4.2 years for patients with cancer and 5.4 years for controls.

**Table 1:** Baseline Characteristics of Patients With Cancer and Matched Cancer-Free Controls by OHCA Status

	Cancer		Control		SMD
	OHCA (n = 8,300)	No OHCA (n = 790,151)	OHCA (n = 36,761)	No OHCA (n = 3,626 181)	
Age at diagnosis/matching date	73.00 [66.00, 79.00]	70.00 [61.00, 78.00]	75.00 [69.00, 81.00]	70.00 [61.00, 78.00]	0.006
Sex					0.015
Male	5,594 (67.4)	417,886 (52.9)	26,449 (71.9)	1,888 739 (52.1)	
Female	2,706 (32.6)	372,265 (47.1)	10,312 (28)	1,737 442 (47.9)	
Unweighted Charlson Comorbidity Index (excluding cancer)					0.050
0	4,667 (56.4)	581,452 (73.6)	18,372 (50.0)	2,625 812 (72.4)	
1-2	3,167 (38.2)	191,776 (24.3)	15,059 (41.0)	897,983 (24.8)	
3-4	428 (5.2)	15,940 (2.0)	2,998 (8.2)	94,294 (2.6)	
≥5	38 (0.5)	983 (0.1)	332 (0.9)	8,092 (0.2)	
Educational level					0.143
Primary	3,106 (39.8)	220,605 (29.8)	15,481 (43.3)	1,052 214 (30.0)	
Secondary	3,165 (40.6)	311,606 (42.1)	13,832 (38.6)	1,458 087 (41.6)	
Postsecondary	1,522 (19.5)	207,230 (28.0)	6,475 (18.1)	994,439 (28.4)	
Income level					0.292
Q1 (lowest)	1,780 (22.4)	135,598 (17.4)	10,006 (27.2)	712,191 (19.7)	
Q2	2,041 (25.7)	154,142 (20.4)	9,812 (26.7)	720,360 (19.9)	
Q3	1,614 (20.3)	151,454 (20.0)	7,169 (19.5)	706,546 (19.5)	
Q4	1,333 (16.8)	154,156 (20.4)	5,479 (14.9)	728,207 (20.1)	
Q5 (highest)	1,175 (14.8)	164,616 (21.8)	4,273 (11.6)	753,334 (20.8)	
Baseline CVD (any)^a^	6,304 (76.0)	472,408 (59.8)	27,877 (75.8)	1,862 516 (51.4)	0.166
Baseline cardiovascular treatment					
Betablocker	4,215 (50.8)	267,480 (33.9)	18,896 (51.4)	1,015 650 (28.0)	
ACE inhibitor/ARB	4,629 (55.8)	326,812 (41.4)	20,181 (54.9)	1,230 725 (33.9)	
Calcium-channel blocker	2,899 (34.9)	212,333 (26.9)	12,590 (34.2)	751,489 (20.7)	
Diuretic agents	3,419 (41.2)	207,324 (26.2)	14,876 (40.5)	743,285 (20.5)	

Values are n (%) or median [IQR]. Standardized mean difference (SMD) was calculated to assess for baseline differences between cancer vs controls, regardless of OHCA status.

ACE = angiotensin-converting enzyme; ARB = angiotensin II receptor blocker; CVD = cardiovascular disease; OHCA= out-of-hospital cardiac arrest.

a Any of baseline CVD (heart failure, ischemic heart disease, arrhythmia, or hypertension) or baseline cardiovascular treatment.

### Risk of OHCA among all cancers combined

Individuals with a cancer diagnosis (all cancers combined) had a higher unadjusted incidence rate of OHCA than controls (Table 2). After multivariable adjustment, cancer was associated with a 55% higher hazard of OHCA than matched controls (Figure 1). In sensitivity analysis excluding cases where cancer diagnosis and OHCA occurred on the same day, the association slightly attenuated but remained robust (HR: 1.47; 95% CI: 1.43-1.51) (Supplemental Figure 3). When stratified by metastasis status, patients with metastatic cancer had an approximately fourfold higher hazard of OHCA than matched controls, whereas those without metastases had a 30% higher hazard (Supplemental Figure 4). The association between cancer diagnosis and OHCA is summarized in the Central Illustration.

**Table 2:** Unadjusted Incidence Rates per 1,000 Person-Years and 95% CIs of OHCA by Cancer Site

Cancer Site	Cancer			Controls		
	Events	Person-Years	IR (95% CI)	Events	Person-Years	IR (95% CI)
All cancers combined	7,595	3,196 945	2.4 (2.3-2.4)	32,859	19,791 999	1.7 (1.6-1.7)
Breast	522	550,054	0.9 (0.9-1)	2,303	2,921 695	0.8 (0.8-0.8)
Colorectal	913	326,126	2.8 (2.6-3.0)	4,222	2,227 399	1.9 (1.8-2.0)
Endocrinological	161	119,378	1.3 (1-1.6)	527	627,903	0.8 (0.8-0.9)
Endometrial	115	70,400	1.6 (1.3-1.9)	385	396,762	1.0 (0.9-1.1)
Gastrointestinal	654	106,465	6.1 (5.7-6.6)	2,804	1,605 070	1.7 (1.7-1.8)
Genitourinary	778	268,043	2.9 (2.7-3.1)	3,271	1,668 115	2.0 (1.9-2.0)
Gynecological, other	159	105,245	1.5 (1.3-1.7)	639	721,663	0.9 (0.8-1.0)
Head and neck	248	76,181	3.3 (2.9-3.7)	862	513,462	1.7 (1.6-1.8)
Leukemia	152	66,154	2.3 (1.9-2.7)	833	493,145	1.7 (1.6-1.8)
Lung	1,031	103,631	9.9 (9.3-10.6)	2,767	1,556 149	1.8 (1.7-1.8)
Lymphoma	254	114,778	2.2 (1.9-2.5)	1,242	756,427	1.6 (1.5-1.7)
Malignant melanoma	319	245,994	1.3 (1.2-1.4)	1895	1,324 861	1.4 (1.4-1.5)
Multiple myeloma	125	36,389	3.4 (2.8-4)	492	274,521	1.8 (1.6-2.0)
Neurological	176	77,206	2.3 (1.9-2.6)	564	575,789	1.0 (0.9-1.1)
Other cancer	247	56,168	4.4 (3.8-4.9)	1,165	708,961	1.6 (1.5-1.7)
Hematological, other	165	58,762	2.8 (2.4-3.2)	682	380,416	1.8 (1.7-1.9)
Prostate	1,519	712,787	2.1 (2-2.2)	8,349	3,620 640	2.3 (2.3-2.4)
Skin	762	280,051	2.7 (2.5-2.9)	3,945	1,536 443	2.6 (2.5-2.6)

For all cancers combined and for each cancer site, events, person-years, and IRs are presented for patients with cancer and their corresponding matched cancer-free controls. Unadjusted incidence rates presented per 1,000 person-years.

IR = incidence rate; other abbreviation as in Table 1.

**Figure 1:**
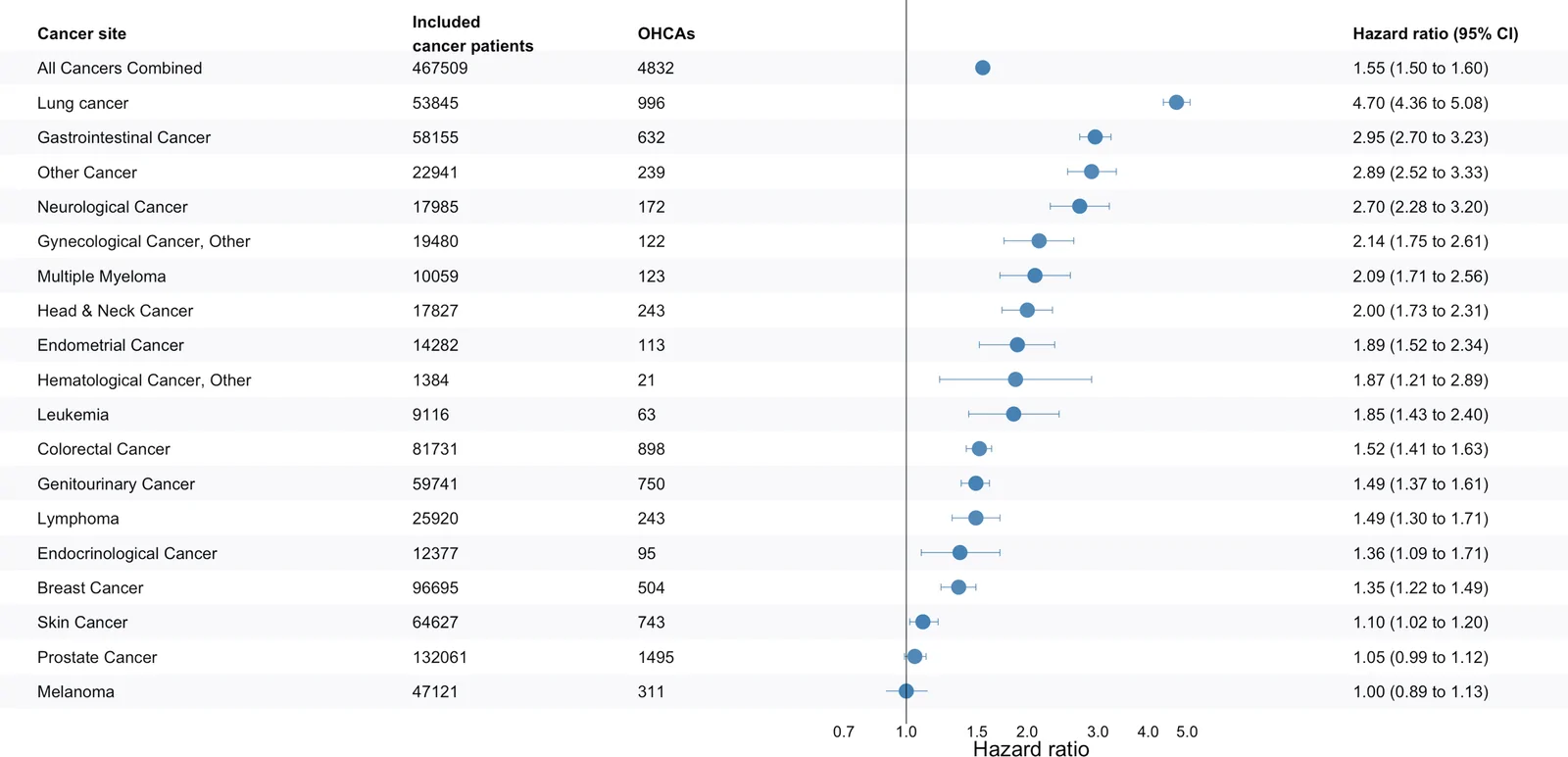
Adjusted Out-of-Hospital Cardiac Arrest Risk by Cancer Status and Site This figure presents HRs with 95% CIs for out-of-hospital cardiac arrest (OHCA) among individuals with cancer diagnosis compared with matched cancer-free controls, overall (all cancers combined) and by cancer site. Associations were estimated using survival analysis (Cox proportional hazards models) adjusted for age at cancer diagnosis or matching date, sex, Charlson Comorbidity Index, baseline cardiovascular disease, year of cancer diagnosis or matching, educational level, and income. *Included cancer patients* denotes the number of patients with each cancer site included in the analyses, *OHCAs* denotes the number of observed OHCAs among these patients during follow-up.

**Central Illustration:**
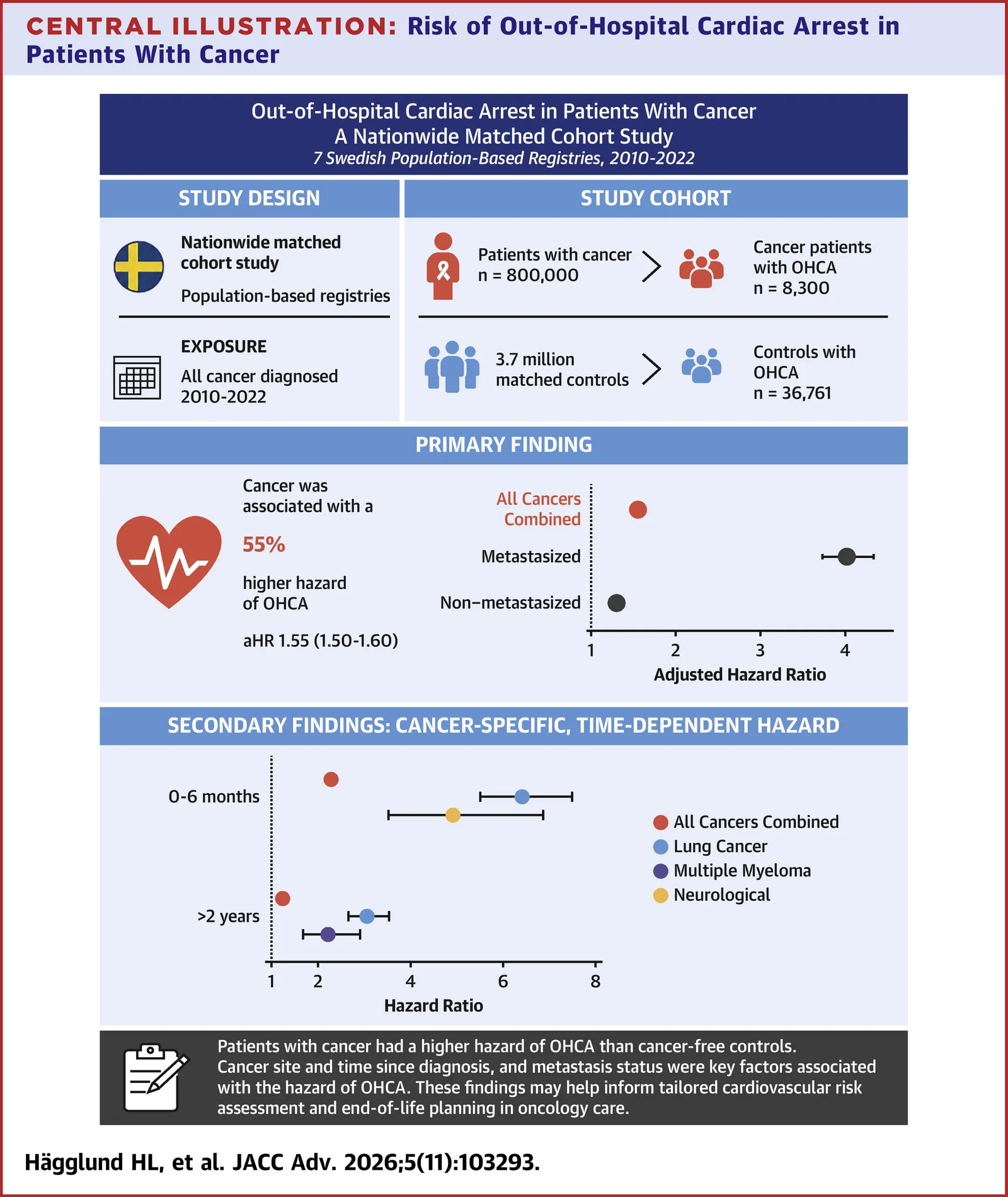
Risk of Out-of-Hospital Cardiac Arrest in Patients With Cancer A total of almost 800,000 cancer patients and 3.7 million matched cancer-free controls were included in this cohort study, based on 7 national, population-based registries. Key findings are shown. Cancer was associated with a 55% increased hazard of out-of-hospital cardiac arrest (OHCA) compared with matched controls. Patients with metastasized cancer had an approximately fourfold increased hazard of OHCA compared with matched controls, whereas those without metastases had an approximately 30% higher hazard. The increased OHCA hazard was greatest during the early phase after cancer diagnosis, although remained increased long term. The greatest short-term risk was seen in patients with lung and neurological cancers, approximately 6-fold compared with controls, and the greatest long-term risk was seen in patients with lung cancer and multiple myeloma, approximately 2- to 3-fold. aHR = adjusted HR.

In cumulative incidence analyses, patients with cancer had a higher cumulative incidence of OHCA than matched controls across all age and sex strata. However, among males and individuals ≥65 years of age, the cumulative incidence curves converged and crossed during follow-up, reflecting the substantial competing risk of death in these groups. Although the relative hazards were elevated, the absolute cumulative incidence of OHCA among patients with cancer remained low, at approximately 1% among females and individuals <65 years of age and approximately 2% among males and individuals ≥65 years of age (Figure 2).

**Figure 2:**
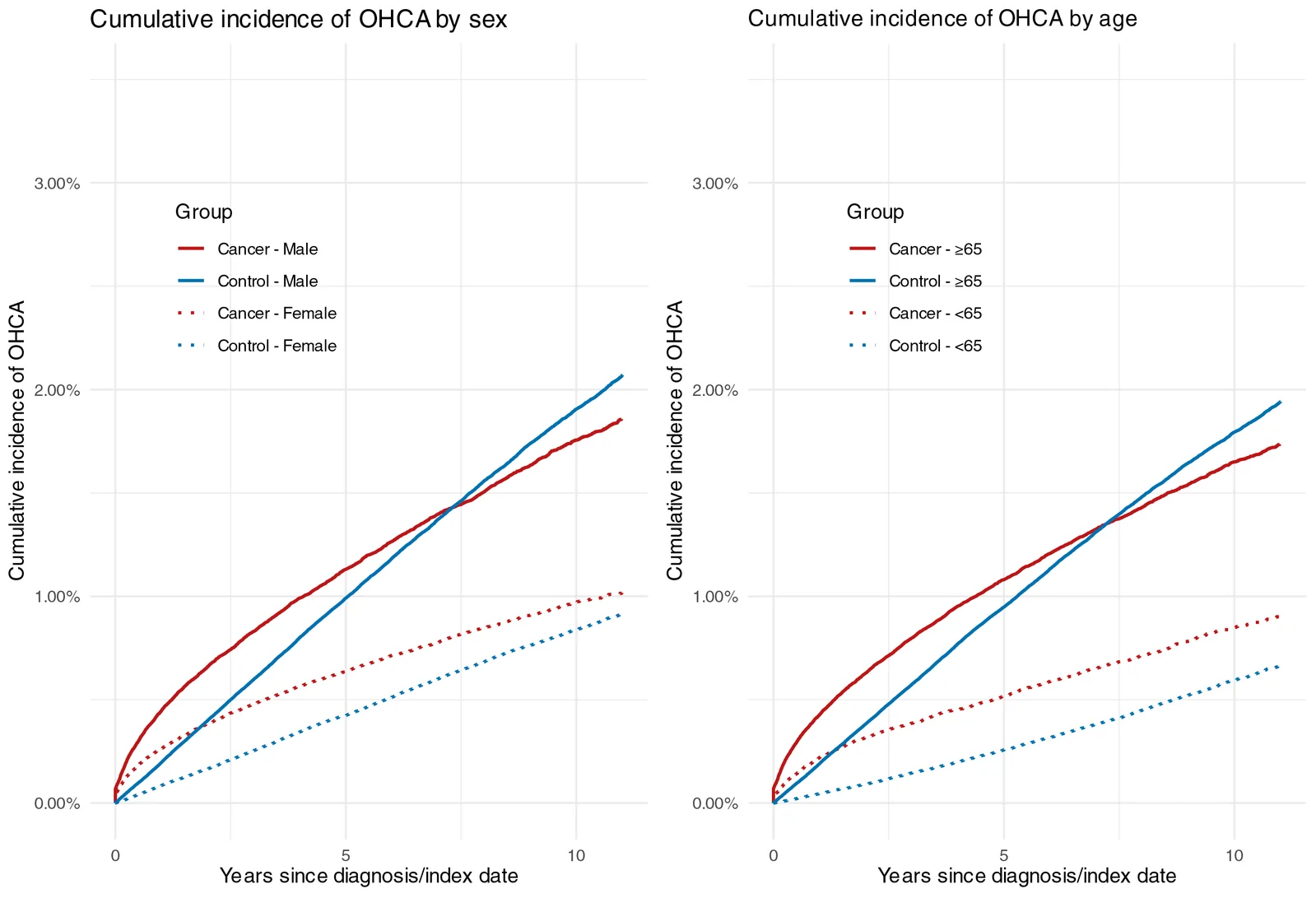
Cumulative Incidence of Out-of-Hospital Cardiac Arrest by Cancer Status, Sex, and Age Cumulative incidence of out-of-hospital cardiac arrest (OHCA) among patients with cancer and matched controls, stratified by sex and age (<65 and ≥ 65 years). Cumulative incidence functions were estimated accounting for the competing risk of death.

### Risk of OHCA by cancer site

The highest risk of OHCA was observed among patients with lung cancer, which was associated with an almost 5-fold higher hazard of OHCA compared with matched controls after multivariable adjustment. Gastrointestinal and neurological cancers were also among the cancer sites associated with the greatest relative risk of OHCA compared with controls (Figure 1). In total, the majority of cancer sites were associated with significantly higher hazards of OHCA after multivariable adjustment, with the exception of malignant melanoma and prostate cancer (Figure 1).

### Risk of OHCA by time since cancer diagnosis

During the first 6 months after cancer diagnosis, 14 of the 18 cancer sites were associated with an increased hazard of OHCA, with gastrointestinal and neurological cancers exceeding a 4-fold increase compared with matched controls (Figure 3). Lung cancer was associated with the highest risk, with a more than 6-fold higher hazard of OHCA during the first year after cancer diagnosis compared with controls. During long-term follow-up, 13 sites remained associated with elevated OHCA hazards, most notably lung cancer, multiple myeloma, and other hematological cancers, which were associated with more than twice the hazard of OHCA compared with controls.

**Figure 3:**
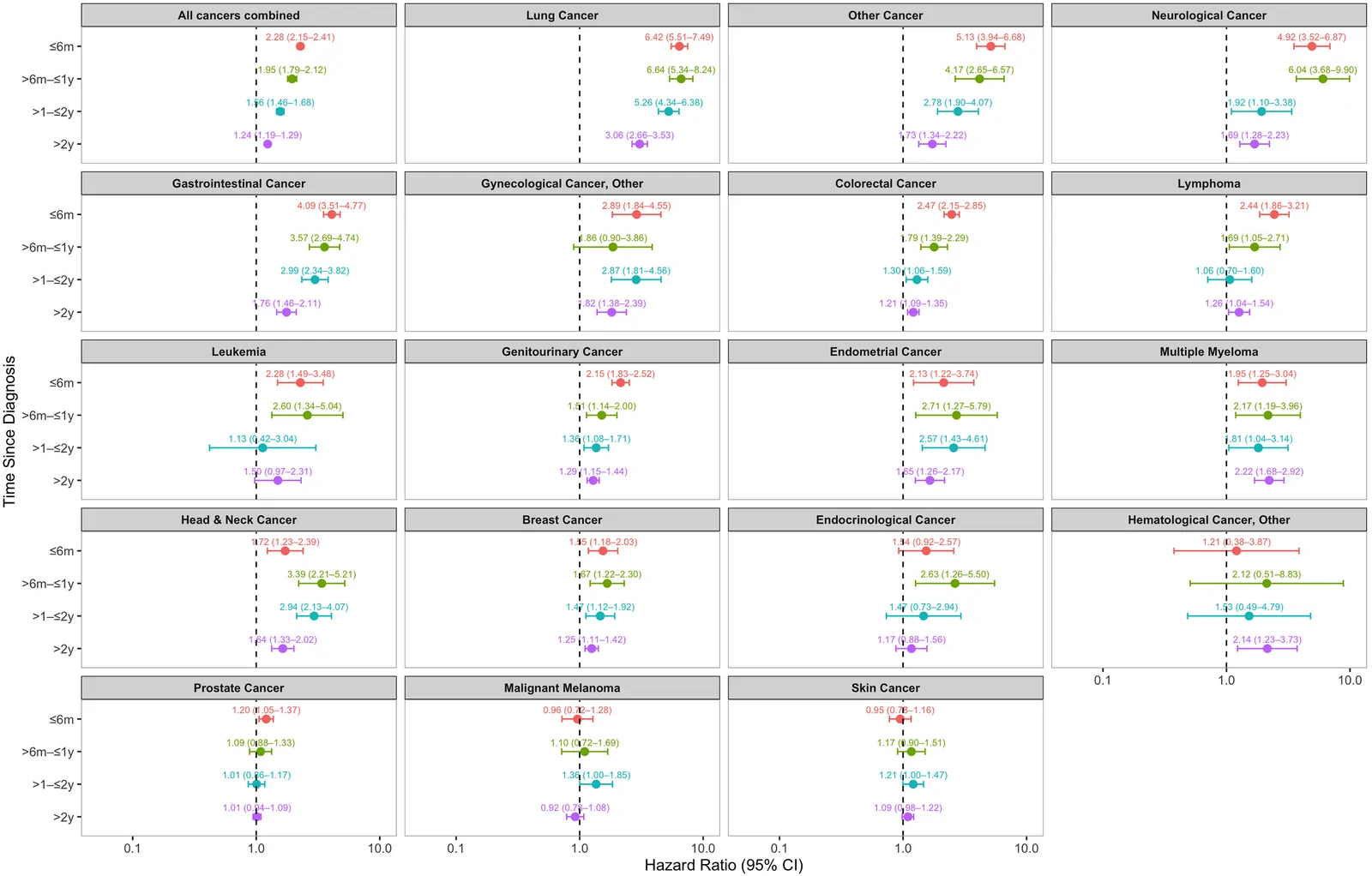
Time-Varying Out-of-Hospital Cardiac Arrest Risk After Cancer Diagnosis This figure presents HRs with 95% CIs for out-of-hospital cardiac arrest (OHCA) by time since cancer diagnosis, for all cancers combined and by cancer site, compared with matched cancer-free controls. Associations were estimated using Cox proportional hazards models adjusted for age at cancer diagnosis/matching date, sex, Charlson Comorbidity Index, baseline cardiovascular disease, year of cancer diagnosis or matching, educational level, and income. HRs are presented for ≤6 months (red), >6 months to ≤1 year (green), >1 to ≤2 years (blue), and >2 years (purple).

The greatest increase in the cumulative incidence of OHCA occurred during the first year after cancer diagnosis (Supplemental Figure 5). The largest increases in cumulative incidence were observed for lung, neurological, gastrointestinal, head and neck, and endometrial cancers, as well as multiple myeloma. Across most cancer sites, cumulative incidence was higher among males and individuals ≥65 years of age at diagnosis or matching. Lung cancer was an exception, with a similar cumulative incidence of OHCA among patients <65 and ≥ 65 years of age.

### OHCA related to cardiovascular disease and cardiovascular risk factors

Among patients with OHCA, those with cancer had a significantly higher baseline prevalence of heart failure, atrial fibrillation, ventricular and other arrhythmias, and hypertension compared with controls, whereas the prevalence of baseline IHD did not differ between the groups (Table 3). Compared with OHCA controls, cancer patients with OHCA had significantly higher hospitalization rates for IHD, atrial fibrillation, other arrhythmias, and hypertension at both 6 months and 1 year after cancer diagnosis, whereas increased hospitalization for heart failure was observed only within the first 6 months and no difference was observed for ventricular arrhythmia. Hypertension showed the greatest difference between cancer patients with OHCA and controls, with both a higher baseline prevalence (26% vs 17%) and a more than 3-fold higher rate of hospitalization for hypertension at 6 months.

**Table 3:** Baseline Cardiovascular Disease and Cardiovascular Hospitalizations by OHCA Status in Cancer Patients and Matched Controls

	Cancer		Controls		*P* Value^a^	*P* Value^b^
	With OHCA	No OHCA	With OHCA	No OHCA		
Baseline CVD						
Heart failure	982 (11.8)	32,929 (4.2)	3,894 (10.6)	104,261 (2.9)	**<0.001**	**0.002**
IHD	1,262 (15.2)	47,834 (6.1)	5,396 (14.7)	178,054 (4.9)	**<0.001**	0.348
Atrial fibrillation	393 (4.7)	26,574 (3.4)	1,112 (3)	82,721 (2.3)	**<0.001**	**<0.001**
Ventricular arrhythmia	224 (2.7)	4,388 (0.6)	765 (2.1)	15,295 (0.4)	**<0.001**	**0.001**
Other arrhythmias	1,193 (14.4)	69,811 (8.8)	4,684 (12.7)	237,404 (6.5)	**<0.001**	**<0.001**
Hypertension	2,128 (25.6)	149,914 (19)	6,053 (16.5)	385,975 (10.6)	**<0.001**	**<0.001**
Hospitalization due to CVD within 6 months after cancer diagnosis or matching						
Heart failure	209 (2.5)	10,025 (1.3)	752 (2)	23,708 (0.7)	**<0.001**	**0.008**
IHD	227 (2.7)	12,163 (1.5)	654 (1.8)	23,891 (0.7)	**<0.001**	**<0.001**
Atrial fibrillation	72 (0.9)	5,663 (0.7)	182 (0.5)	10,030 (0.3)	0.123	**<0.001**
Ventricular arrhythmia	7 (0.1)	379 (0)	26 (0.1)	1,036 (0)	0.228	0.879
Other arrhythmias	266 (3.2)	18,247 (2.3)	629 (1.7)	28,720 (0.8)	**<0.001**	**<0.001**
Hypertension	652 (7.9)	61,966 (7.8)	799 (2.2)	49,642 (1.4)	0.636	**<0.001**
Hospitalization due to CVD during 1st year after cancer diagnosis or matching						
Heart failure	318 (3.8)	14,687 (1.9)	1,336 (3.6)	42,093 (1.2)	**<0.001**	0.473
IHD	317 (3.8)	16,399 (2.1)	1,175 (3.2)	43,746 (1.2)	**<0.001**	**0.005**
Atrial fibrillation	107 (1.3)	7,859 (1)	326 (0.9)	18,532 (0.5)	**0.010**	**0.001**
Ventricular arrhythmia	11 (0.1)	587 (0.1)	56 (0.2)	1,929 (0.1)	0.094	0.752
Other arrhythmias	375 (4.5)	24,275 (3.1)	1,124 (3.1)	52,728 (1.5)	**<0.001**	**<0.001**
Hypertension	783 (9.4)	73,009 (9.2)	1,513 (4.1)	93,767 (2.6)	0.334	**<0.001**

Values are n (%). **Bold** values indicates statistically significant *P* values (*P* < 0.05).

IHD = ischemic heart disease; other abbreviations as in Table 1.

a Chi-square test comparing cancer patients with OHCA and those without OHCA.

b Chi-square test comparing cancer patients with OHCA and matched cancer-free controls with OHCA.

Among patients with cancer, those with OHCA had a greater burden of baseline CVD, and higher rates of hospitalization due to heart failure, IHD, and other arrhythmias than those without OHCA (Table 3). In stratified analyses, the association between cancer and OHCA varied by age at diagnosis or matching, sex, and baseline CVD status, with higher cause-specific HRs observed among younger individuals, women, and those without baseline CVD (Supplemental Table 6). Among patients with a CVD diagnosis at baseline, patients with cancer who experienced OHCA had a similar prevalence of cardiovascular treatment compared with controls with OHCA (Supplemental Table 7).

All cardiovascular risk factor–related cancer groups were associated with a higher OHCA risk than matched controls. Diet- and smoking-related cancers showed the strongest associations, each with more than a 2-fold higher hazard of OHCA (Supplemental Figure 6).

## Discussion

This nationwide study of nearly 800,000 patients with cancer and 3.7 million matched controls is, to our knowledge, the largest population-based study to investigate the risk of OHCA across a broad spectrum of cancer sites. We found a 55 percent higher hazard of OHCA among patients with cancer than among controls, after adjustment for baseline risk factors. Cancers of the lung, gastrointestinal tract, and nervous system were associated with particularly high risks of OHCA. The excess hazard of OHCA was greatest during the first year after cancer diagnosis but remained elevated throughout long-term follow-up. These findings may help inform individualized cardiovascular risk assessment among patients with cancer.

### Factors associated with OHCA among patients with cancer

Among individuals who experienced an OHCA in our study, patients with cancer were younger, had a lower comorbidity burden, and had more favorable socioeconomic factors than matched controls. Consistent with these findings, the relative association between cancer and OHCA was stronger among younger individuals and those without baseline CVD, suggesting that factors beyond traditional cardiovascular risk factors contribute to OHCA among patients with cancer. In line with previous studies,^3^^,^^33^, ^34^, ^35^, ^36^ we found a greater burden of baseline CVD and hospitalization due to CVD among cancer patients compared with controls. However, the increased burden of CVD cannot fully explain the increased risk of OHCA among cancer patients, as this was adjusted for in the multivariable analyses. Furthermore, noncardiac causes of OHCA, including pulmonary embolism, hemorrhage, and hypoxia, appear to be more common among patients with cancer^37^, ^38^, ^39^, ^40^ and may contribute to the higher incidence of OHCA in this population.

### Clinical and temporal variation in OHCA risk

In our study, patients with metastatic cancer had a substantially greater hazard of OHCA than matched controls. Previous studies have similarly reported increased cardiovascular risk among patients with metastatic cancer,^41^ and this association may be influenced by factors such as a higher burden of systemic inflammation, more intensive cancer treatment, and a greater prevalence of cancer-related complications.

The cancer sites associated with the greatest risk of OHCA in our study are largely concordant with findings from prior studies^3^^,^^42^^,^^43^ of CVD and cardiovascular mortality among patients with cancer. Elevated risks for lung/thoracic,^3^^,^^42^^,^^43^ gastrointestinal,^42^ neurological,^42^ hematological,^3^^,^^42^^,^^43^ gynecological,^3^ and head and neck^43^ cancers have been consistently reported previously and are consistent with our findings. In a previous study of 175,000 patients with newly diagnosed cancer,^44^ the cancer sites associated with an increased risk of OHCA were largely similar to those identified in our study. Taken together, although site-specific patterns of OHCA risk are evident in our study, the mechanisms underlying the observed associations between cancer and OHCA remain incompletely understood.

Our finding that the risk of OHCA was greatest during the first year after cancer diagnosis and remained elevated thereafter is consistent with previous studies of CVD among patients with cancer.^41^^,^^43^^,^^45^ The early excess risk may be associated with cardiovascular complications occurring in the context of the cancer and its treatment. In our study, long-term follow-up was defined as >2 years after cancer diagnosis, due to a lack of data on the time of cancer treatment. Although this definition differs from that of the European Society of Cardiology cardio-oncology guidelines, which define events as occurring “beyond 12 months after completing cardiotoxic treatment”,^1^ the 2 definitions are likely to substantially overlap, as many patients complete primary therapy within the first year following diagnosis.

Site-specific differences in OHCA risk according to time since cancer diagnosis may reflect differences in baseline cardiovascular risk, cancer biology, and timing of treatment-related cardiotoxicity. Lung cancer and multiple myeloma are frequently treated with systemic therapies that have been associated with both early and late cardiovascular toxicity, which may partly explain the persistently elevated risk observed.^1^^,^^46^, ^47^, ^48^, ^49^, ^50^ For lung cancer, in addition to shared risk factors and cardiotoxic treatments, the tumor’s anatomical location may itself contribute to cardiorespiratory complications.^51^, ^52^, ^53^ Gastrointestinal cancers, particularly pancreatic and gastric cancer, are strongly associated with cardiometabolic risk factors and hypercoagulability, as well as exposure to potentially cardiotoxic chemotherapy.^1^^,^^54^, ^55^, ^56^, ^57^ Although neurological cancers are heterogeneous with respect to treatment and cardiovascular risk, patients with central nervous system tumors have been reported to have an increased risk of CVD.^58^^,^^59^

Several lifestyle factors, including smoking, diet, obesity, heavy alcohol consumption, and physical activity, are associated with the risk of both CVD and cancer.^60^ In exploratory analyses, we found that all cancer groups defined according to shared cardiovascular risk factor-related etiological characteristics were associated with an increased hazard of OHCA. Diet- and smoking-related cancers showed the strongest associations. Notably, both groups included lung cancer, the cancer site associated with the highest OHCA hazard in the present study, raising the possibility that shared cardiovascular risk factors may be associated with the observed patterns of OHCA risk.

Although patients with cancer had substantially higher hazards of OHCA than matched controls, the absolute risk remained low. Nevertheless, given the poor prognosis of OHCA, with survival probabilities of approximately 10%^61^ and potentially even lower among patients with cancer,^10^ the observed differences in cumulative incidence among high-risk groups may still be clinically meaningful.

Interpretation of our findings should take into account that the OHCA definition used focuses on events among patients for whom resuscitation was initiated or continued, thereby emphasizing the clinical relevance of the included cases. In the *Swedish Register of Cardiopulmonary Resuscitation*, OHCA cases are registered only when cardiopulmonary resuscitation is initiated or continued by EMS. Consequently, cardiac arrests occurring among patients with cancer who are hospitalized with advanced disease, receiving palliative care, or have a do-not-resuscitate decision (eg in hospice settings) are not captured. Thus, the included cancer population experiencing an OHCA likely reflects patients in whom survival was considered a realistic and appropriate goal of care. This selection may result in an underestimation of the true incidence of OHCA among patients with cancer overall and may introduce bias if resuscitation practices differ systematically between patients with and without cancer.

### Clinical Implications

Cardiovascular complications are a growing concern among patients with cancer, underscoring the need for a multidisciplinary approach that considers patients’ overall health. Current cardio-oncology guidelines provide recommendations for cardiovascular care in patients with cancer.^1^ However, cardio-oncology remains a relatively new field, and further research is needed to strengthen the evidence supporting current recommendations.^1^ In this context, the present study may contribute to risk stratification by identifying cancer sites associated with particularly high OHCA risk, especially during the first year after cancer diagnosis. These findings may help inform individualized cardiovascular risk assessment, preventive strategies, shared decision-making, and end-of-life planning.

### Strengths and limitations

Key strengths of our study include the large sample size, long follow-up period, and the comprehensive national registers included, which enabled subgroup analyses and extensive adjustment for potential confounders. Importantly, the study includes all patients diagnosed with cancer and all OHCA cases identified in Sweden. There were 3 main limitations of the study. First, cancer treatment and progression were not considered, as these data were not available in the included registers. Second, individual lifestyle risk factors were not included due to a lack of data and could not be directly accounted for in the analyses. However, adjustments were made for socioeconomic factors, which are associated with many lifestyle-related risk factors. Furthermore, exploratory analyses examining OHCA hazards across cancer groups defined by shared cardiovascular risk factor-related etiological characteristics were performed to assess whether patterns differed according to cardiovascular risk factor profiles. Third, nonspecialized outpatient cardiovascular diagnoses were not captured, which may cause an underestimation of cardiovascular burden; however, cardiovascular treatment is included as a proxy for this. Lastly, many analyses were conducted in addition to the primary analyses; these should be considered exploratory and interpreted with caution. In addition, for some cancer groups, such as “other hematological cancer”, the small number of OHCAs resulted in wide CIs, warranting cautious interpretation of the estimates. Nevertheless, this study is, to our knowledge, the first nationwide and globally the largest, most comprehensive study of OHCA risk among cancer patients.

## Conclusions

In our study, cancer was associated with an increased hazard of OHCA compared with matched cancer-free controls, with the greatest excess risk observed among patients with lung, gastrointestinal tract, and neurological cancers. The association was most pronounced within the first year after cancer diagnosis, highlighting a period of increased OHCA occurrence that may warrant attention in clinical risk assessment. Our findings identify cancer site, time since diagnosis, and metastasis status as key factors associated with the hazard of OHCA. Although additional work is needed to clarify the influence of cancer therapies and other modifiable factors, our findings provide clinically relevant insights into high-risk profiles that may help inform clinical risk assessment, preventive strategies, monitoring, as well as shared decision-making and end-of-life planning.

Perspectives **COMPETENCY IN MEDICAL KNOWLEDGE:** Cancer is associated with an increased risk of OHCA with controls, particularly during the first year after diagnosis and varying by cancer site.

**TRANSLATIONAL OUTLOOK:** Additional work is needed to clarify the influence of cancer therapies and other modifiable factors on the risk of developing OHCA among patients with cancer.

## Funding support and author disclosures

This work was supported by the Stig Holmberg Grant from the Foundation for Cardiopulmonary Resuscitation in Sweden, the Swedish Resuscitation Council, and the Serafimer Hospital Foundation. Further the study was funded by the Stig Holmberg Grant of the Foundation for cardio-pulmonary resuscitation in Sweden (grant number 2023-84, 2025-109); Project Grant from the Swedish Resuscitation Council; and The Serafimer Hospital Foundation Research Grant. Dr Hägglund is a member (unpaid) of the working group for CardioOncology of the Swedish Association of Cardiology. Dr Månsson Broberg has received grants from Swedish Research Council, Center for Innovative Medicine, Stockholm Region, and Swedish Childhood Cancer Foundation; has received royalites/licenses for writing chapter about cardio-oncology in textbook about women’s cardiovascular health, and payment/honorary from Pfizer, BMS, Orion, ipsen, and AstraZeneca (payed to Karolinska University Hospital); is chair of working group for CardioOncology of the Swedish Association of Cardiology; and a board member of the Nordic CardioOncology Society. Dr Jonsson has received grant from Swedish Heart-Lung Foundation. Dr Hedayati is a board member (unpaid) of True Dose AB (co-founder) and All Cancer Sverige. All other authors have reported that they have no relationships relevant to the contents of this paper to disclose.
