# Supplementary material for: Increased Out-of-Hospital Cardiac Arrest Risk in Cancer: Evidence From 800,000 Patients and 3.7 Million Controls
Source: JACC Adv. 2026 Oct 5;5(11):103293. doi: 10.1016/j.jacadv.2026.103293 (PMC13671319; doi:10.1016/j.jacadv.2026.103293)
Supplement: Supplementary file 1 [file mmc1.docx]

# Supplemental Appendix

## Supplemental Tables

| **Supplemental Table 1 –** Distribution of diagnoses by cancer site category | | |
| --- | --- | --- |
| **Cancer site** | **ICD-code** | **Number of cases, n (%)** |
| Breast | C50 | Breast 100024 (100%) |
| Colorectal | Colon (C18), rectum (C19–C20) | Colon 57274 (69%), rectal 26315 (31%) |
| Endocrinological | Thyroid (C73), other endocrinological (C74-C75) | Thyroid 7623 (36%), other endocrinological 13377 (64%) |
| Endometrial | C541 | Endometrial 14662 (100%) |
| Gastrointestinal | Pancreatic (C25), Liver (C22), gastric (C16), esophagus (C15), gall bladder (C23), other parts of biliary tract (C24), anus (C21), small intestine (C17), other (C26) | Pancreatic 18559 (31%), gastric 11161 (19%), liver 9837 (16%), esophagus 6388 (11%), small intestine 4180 (7%), other parts of biliary tract 3590 (6%), gall bladder 2506 (4%), anus 2527 (4%), other 1096 (2%) |
| Genitourinary | Male genital (C60, C62-C63), kidney (C64), upper urinary tract (C65-C66), bladder (C67), other urinary (C68) | Male genital 6255 (10%), kidney 15736 (25%), upper urinary tract 3312 (5%), bladder 36224 (59%), other urinary 454 (0.7%) |
| Gynecological, other | Ovarian (C56), cervical (C53), vulva (C51), uterus (C540, C542–C543, C548–C549, C559), other (C57, C589), vagina (C52) | Ovarian 7185 (31%), Cervical 6452 (27%), Vulva 2408 (10%), uterus 5043 (21%), other 2084 (9%), vagina 359 (2%) |
| Head- and neck | Oral (C00, C02–C06), oropharynx (C01, C09-C10), hypopharynx (C12-C13), nasopharynx (C11), larynx (C32), salivary glands (C07–C08), nasal/sinuses (C30-C31), other (C14) | Oral 7571 (41%), oropharynx 5151 (28%), hypopharynx 709 (4%), nasopharynx 422 (2%), larynx 2187 (12%), salivary glands 1426 (8%), nasal/sinuses 917 (5%), other 50 (0.3%) |
| Leukemia | Lymphoid (C91), myeloid (C92), other leukemias (C93-C95) | Lymphoid 9683 (55%), myeloid 7324 (42%), other leukemias 631 (4%) |
| Lung | C34 | 55237 (100%) |
| Lymphoma | Hodkin’s lymphoma (C81), non-Hodgkin’s lymphoma (C82–C85) | Hodgkin’s lymphoma 2658 (10%), non-Hodkin’s lymphoma 24613 (90%) |
| Malignant melanoma | C43 | Malignant melanoma 48049 (100%) |
| Multiple myeloma | C90 | Multiple Myeloma 10290 (100%) |
| Neurological | Brain (C71), CNS meninges (C70), spinal cord, cranial nerve, and other CNS (C72) | Brain 9839 (52%), CNS meninges 6969 (37%), spinal cord, cranial nerve, and other CNS 2064 (11%) |
| Other | Thoracic (excluding lung) (  C33, C37-C39), Bone and soft tissue (C40–C41, C45-C49), Eye (C69), Other, ill-defined sites, secondary, unknown (C76–C80) | Thoracic (excluding lung) 2524 (11%), bone and soft tissue 5997 (25%), eye 1746 (7%), other, ill-defined sites, secondary, unknown 13479 (57%) |
| Hematological, other | Malignant immunoproliferative diseases (C88), other and unspecified malignant neoplasms of lymphoid, hematopoietic and related tissue (C96), neoplasms of uncertain or unknown behavior (D45-D47) | Malignant immunoproliferative diseases 1320 (9.2%), other and unspecified malignant neoplasms of lymphoid, hematopoetic and related tissue 96 (0.7%), neoplasms of uncertain or unknown behavior 12861 (90%) |
| Prostate | C61 | Prostate 134051 (100%) |
| Skin | C44 | Skin 65 914 (100%) |
| Distribution of diagnoses by cancer site category as number and percentages. Cancer site categories represent composite groups used throughout the manuscript. ICD, international classification of disease | | |

| **Supplemental Table 2 –** ICD-codes and included diagnoses per cardiovascular disease, and ATC-codes and included drugs per cardiovascular treatment category | | |
| --- | --- | --- |
| **Cardiovascular disease** | **ICD-10 codes** | **Diagnoses** |
| Heart failure | I110, I130, I132, I255, I420, I426, I427, I428, I429, I43, I50 | Hypertensive heart disease, Hypertensive heart and kidney disease, Hypertensive heart and kidney disease with both (congestive) heart failure and kidney failure, Ischemic cardiomyopathy, Dilated cardiomyopathy, Alcoholic cardiomyopathy, Cardiomyopathy due to drugs and other external agents, other cardiomyopathies, Cardiomyopathy, unspecified, Cardiomyopathy in infectious and parasitic diseases classified elsewhere, Congestive heart failure |
| Ischemic heart disease | I20 - I25 | Angina, acute myocardial infarction, reinfarction, complications of myocardial infarction (hemopericardium, septum defects, rupture of cardiac wall/chordae tendineae/papillar muscle, thrombosis in the heart), coronary thrombosis, post infarction syndrome, chronic ischemic heart disease |
| Atrial fibrillation | I480, I481, I482 | Paroxysmal atrial fibrillation, persistent atrial fibrillation, chronic atrial fibrillation |
| Ventricular tachycardia | I472, I490, I493 | Paroxysmal ventricular tachycardia, Ventricular fibrillation and ventricular flutter, premature depolarization of ventricle |
| Other arrythmias | I44, I45, I470, I471, I479, I483, I484, I489, I491, I492, I494, I495, I498, I499 | Atrioventricular blockage, left anterior fascicular block, left posterior fascicular block, left bundle branch block, right fascicular block, right bundle-branch block, bifascicular block, trifascicular block, unspecified intraventricular block, other specified heart block, pre-excitation syndrome, conduction disorder unspecified, re-entry ventricular arrythmia, paroxysmal supraventricular tachycardia, paroxysmal tachycardia unspecified, typical atrial flutter, atypical atrial flutter, atrial fibrillation and atrial flutter unspecified, premature atrial depolarization, premature junctional depolarization, other and unspecified premature depolarization, sick sinus syndrome, other specified cardiac arrythmias, cardiac arrythmia unspecified |
| Hypertension | I10 - I15 | Essential hypertension, hypertensive heart disease, hypertensive renal disease, hypertensive heart and renal disease, secondary hypertension |
| **CV treatment** | **ATC-code** | **Treatment** |
| Betablocker | C07 | Non-selective beta-blockers (Propanolol, Sotalol), Selective beta-blockers (Metoprolol, Atenolol, Bisoprolol, Esmolol, Landiolol), Alfa- and beta-receptor blockers (Labetolol, Carvedilol), combined Metoprolol and Felodipine |
| ACE inhibitors/ARB | C09 | ACE inhibitors (Kaptopril, Enalapril, Lisinopril, Perindopril, Ramipril), combined ACE inhibitor and diuretics (i.e. Enalapril comp), Angiotensin-receptor blockers (Losartan, Valsartan, Irbesartan, Kandesartan, Telmisartan), combined ARB and diuretics (i.e. Losarstad comp), combined valsartan and amlodipin, combined valsartan and sacubitril (Entresto) |
| Calcium Channel Blocker | C08 | Calcium channel blockers with predominantly vascular selectivity (Dihydropyridine derivatives [i.e amlodipine, felodipine], Other selective calcium channel blockers with mainly vascular effects), **Calcium channel blockers with direct cardiac selectivity (**Phenylalkylamine derivatives [i.e. Verapamil], Benzothiazepine derivatives), non-selective calcium channel blockers (Phenylalkylamine derivatives, Other non-selective calcium channel blockers), Calcium channel blockers and diuretics |
| Diuretics | C03 | Thiazides (Bendroflumethiazide, hydrochlorothiazide, combined Bendroflumethiazide and potassium), Thiazide-related diuretics (Sulfonamide derivatives, Sulfonamides in combination with potassium, Mercurial diuretics, Xanthine derivatives, Sulfonamides in combination with other agents, Other thiazide-related diuretics), Loop-diuretics (Sulfonamides), Aldosterone antagonists, Other potassium-sparing agents, Thiazide-related diuretics and potassium-sparing agents, Vasopressin antagonists |
| ACE, angiotensin-converting enzyme; ARB, angiotensin II receptor blocker; ATC, anatomic therapeutical chemical (classification system); CV, cardiovascular; CVD, cardiovascular disease; ICD-10, international classification of diseases, 10^th^ revision | | |

| **Supplemental table 3 -** Baseline characteristics for patients with cancer compared to matched cancer-free controls | | | |
| --- | --- | --- | --- |
|  | **Cancer** | **Control** | Missing data |
|  | n = 798 451 | n = 3 662 942 |  |
| **Age^a^** | 70.00 [62.00, 78.00] | 70.00 [61.00, 78.00] | 0% |
| **Sex** |  |  | 0% |
| Male | 423 480 (53.0) | 1 915 188 (52.3) |  |
| Female | 374 971 (47.0) | 1 747 754 (47.7) |  |
| **Charlson Comorbidity Index** | | | 0% |
| 0 | 586 119 (73.4) | 2 644 184 (72.2) |  |
| 1-2 | 194 943 (24.4) | 9 130 42 (24.9) |  |
| 3-4 | 16 368 (2.0) | 97 292 (2.7) |  |
| ≥5 | 1 021 (0.1) | 8 424 (0.2) |  |
| **Educational Level** | | | 3.9% |
| Primary | 223 711 (28) | 1 067 695 (29.1) |  |
| Secondary | 314 771 (39.4) | 1 471 919 (40.2) |  |
| Post-Secondary | 208 752 (26.1) | 1 000 824 (27.3) |  |
| **Income Level** | | | 0.9% |
| Q1 (lowest) | 133 378 (16.7) | 722 298 (19.7) |  |
| Q2 | 156 183 (19.6) | 730 172 (19.9) |  |
| Q3 | 153 068 (19.2) | 713 715 (19.5) |  |
| Q4 | 155 489 (19.5) | 733 686 (20.0) |  |
| Q5 (highest) | 165 791 (20.8) | 757 607 (20.7) |  |
| **Baseline CVD (any)^b^** | 477 958 (59.9) | 1 891 592 (51.6) | 0% |
| Hypertension | 152 042 (19.0) | 528 775 (14.4) | 0% |
| Heart Failure | 33 699 (4.2) | 126 285 (3.4) | 0% |
| IHD | 49 096 (6.1) | 199 215 (5.4) | 0% |
| Arrythmia | 76 312 (9.6) | 283 724 (7.7) | 0% |
| **Baseline CV treatment** | | |  |
| Betablocker | 271 695 (34.0) | 1 034 546 (28.2) | 0% |
| ACEi/ARB | 331 441 (41.5) | 1 250 906 (34.2) | 0% |
| CCB | 215 232 (27.0) | 764 079 (20.9) | 0% |
| Diuretics | 210 743 (26.4) | 758 161 (20.7) |  |
| **Metastasis status** | | | 26.4%^c^ |
| No metastasis | 501 573 (62.8) | - |  |
| Metastasis | 86 369 (10.8) | - |  |
| a. Age at cancer diagnosis/matching date. b. Any of baseline CVD or baseline CV treatment. c. Missing metastasis data among patients diagnosed with cancer.  ACE, angiotensin-converting enzyme; ARB, angiotensin II receptor blocker; CCB, calcium channel blocker; CV, cardiovascular; CVD, cardiovascular disease. | | | |

| **Supplemental Table 4 –** Classification of cancer sites into cardiovascular risk factor-related cancer groups | | |
| --- | --- | --- |
| **Cardiovascular risk factor-related cancer group** | **Included cancers (ICD-10 codes)** | **No. of patients** |
| Smoking-related cancers | Oral cavity (not base of tongue) (C00, C02-C06), oropharynx (C10), Nasopharynx (C11), Other pharynx (C09, C12-C14), Esophagus (C15), Stomach (C16), Colon (C18), Rectum (C19-C20), Liver (C22), Pancreas (C25), Nasal cavity and middle ear (C30), paranasal sinuses (C31), Larynx (C32), Lung (including trachea and bronchi (C33-C34), Ovary (C56), Kidney (C64), Urinary bladder (C67), Myeloid leukemia (C92) | 259,315 |
| Weight-related cancers | Esophagus (C15), Stomach (C16), Colon (C18), Rectum (C19-C20), Liver (C22), Gallbladder (C23), Pancreas (C25), Breast (C50), Uterus (54), Ovary (C56), Kidney (C64) | 266,821 |
| Physical inactivity-related cancers | Colon (C18), Rectum (C19-C20), Breast (C50), Uterus (54) | 198,715 |
| Diet-related cancers | Esophagus (C15), Colon (C18), Rectum (C19-C20), Lung (including trachea and bronchi (C33-C34) | 143,895 |
| Alcohol-related cancers | Oral cavity (C00-C06), oropharynx (C10), Nasopharynx (C11), Other pharynx (C09, C12-C14), Esophagus (C15), Colon (C18), Rectum (C19-C20), Liver (C22), Larynx (C32), Breast (C50) | 213,193 |
| Cancer sites were classified according to shared cardiovascular risk factor-related etiological groups. Patients with cancers associated with more than one risk factor were included in each relevant group.  ICD-10, international classification of diseases, 10^th^ revision. | | |

| **Supplemental table 5 –** Distribution of cancer sites | | | | | | |
| --- | --- | --- | --- | --- | --- | --- |
|  | Overall | | Male | | Female | |
|  | n | % | n | % | n | % |
| All cancers combined | 798 451 | 100 | 423 576 | 100 | 375 013 | 100 |
| *Prostate* | 134 051 | 16.8 | 134 051 | 31.7 | NA | NA |
| *Breast* | 100 024 | 12.5 | 666 | 0.2 | 99 358 | 26.5 |
| Colorectal | 83 589 | 10.5 | 44 000 | 10.4 | 39 589 | 10.6 |
| Skin | 65 914 | 8.3 | 36 407 | 8.6 | 29 507 | 7.9 |
| Genitourinary | 61 979 | 7.8 | 45 820 | 10.8 | 16 159 | 4.3 |
| Gastrointestinal | 59 844 | 7.5 | 33 679 | 8 | 26 165 | 7.0 |
| Lung | 55 237 | 6.9 | 26 246 | 6.2 | 28 991 | 7.7 |
| Melanoma | 48 049 | 6.0 | 24 569 | 5.8 | 23 480 | 6.3 |
| Lymphoma | 27 271 | 3.4 | 15 404 | 3.6 | 11 867 | 3.2 |
| Other Cancer | 23 790 | 3.0 | 11 277 | 2.7 | 12 513 | 3.3 |
| *Gynecological, other* | 23 531 | 3.0 | 2 | 0.0 | 23 529 | 6.2 |
| Endocrinological | 21 002 | 2.6 | 6 799 | 1.6 | 14 201 | 3.8 |
| Neurological | 18 872 | 2.4 | 8 907 | 2.1 | 9 965 | 2.7 |
| Head and neck | 18 433 | 2.3 | 11 634 | 2.8 | 6 800 | 1.8 |
| Leukemia | 17 638 | 2.2 | 10 482 | 2.5 | 7 156 | 1.9 |
| *Endometrial* | 14 662 | 1.8 | NA | NA | 14 662 | 4.0 |
| Hematological, other | 14 277 | 1.8 | 7561 | 1.8 | 6 716 | 1.8 |
| Multiple Myeloma | 10 290 | 1.3 | 5 976 | 1.4 | 4 314 | 1.2 |
| Numbers and percentages of included patients per cancer site, for patients overall (both sexes), males, and females. Italic type, cancer sites regarded as sex-specific, diagnoses among other sex are excluded from analyses. | | | | | | |

| **Supplemental table 6 –** Hazard ratios for OHCA comparing cancer patients with matched controls, stratified by baseline characteristics. | | |
| --- | --- | --- |
|  | HR (CI) | p-value interaction term |
| **Age** |  | <0.001 |
| Age <65 years | 2.0 (1.78-2.24) |  |
| Age ≥65 years | 1.53 (1.47-1.59) |  |
| **Sex** |  | <0.001 |
| Female | 1.49 (1.43-1.56) |  |
| Male | 1.9 (1.75-2.07) |  |
| **CCI** |  | 0.129 |
| 0 | 1.57 (1.49-1.65) |  |
| 1-2 | 1.64 (1.54-1.75) |  |
| 3-4 | 1.68 (1.41-2.0) |  |
| ≥5 | 2.01 (1.02-3.98) |  |
| **Education** |  | 0.45 |
| Primary | 1.58 (1.49-1.68) |  |
| Secondary | 1.63 (1.53-1.74) |  |
| Post-secondary | 1.58 (1.45-1.73) |  |
| **Income (quintile)** |  | 0.287 |
| Q1 | 1.62 (1.49-1.77) |  |
| Q2 | 1.56 (1.44-1.68) |  |
| Q3 | 1.56 (1.44-1.70) |  |
| Q4 | 1.63 (1.48-1.80) |  |
| Q5 | 1.68 (1.51-1.88) |  |
| **Hypertension** |  | <0.001 |
| No | 1.65 (1.58-1.73) |  |
| Yes | 1.39 (1.30-1.49) |  |
| **Heart failure** |  | <0.001 |
| No | 1.6 (1.54-1.67) |  |
| Yes | 1.32 (1.2-1.45) |  |
| **Ischemic heart disease** |  | <0.001 |
| No | 1.62 (1.55-1.69) |  |
| Yes | 1.35 (1.24-1.47) |  |
| **Arrythmia** |  | <0.001 |
| No | 1.61 (1.54-1.68) |  |
| Yes | 1.44 (1.32-1.57) |  |
| Interaction analysis where hazard ratios represent comparisons of patients with a cancer diagnosis vs matched controls within each stratum of the interaction term. P-values for interaction were derived from Wald tests.  CCI, Charlson comorbidity Index; CI, confidence interval; HR, hazard ration; OHCA, out-of-hospital cardiac arrest | | |

| **Supplementary Table 7 –** Prevalence of cardiovascular treatment among patients with baseline cardiovascular disease, by OHCA status. | | | | |
| --- | --- | --- | --- | --- |
|  | **Patients With Cancer and Cardiovascular Disease** | | **Controls with Cardiovascular Disease** | |
| Treatment | **No OHCA** | **With OHCA** | **No OHCA** | **With OHCA** |
|  | n = 219 073 | n = 3 558 | n = 964 405 | n = 18 173 |
| Diuretics | 112 076 (51.2%) | 2 183 (61.4%) | 434 781 (45.1%) | 10 943 (60.2%) |
| Betablockers | 151 346 (69.1%) | 2 761 (77.6%) | 621 536 (64.4%) | 14 176 (78%) |
| Calcium channel blocker | 107 008 (48.8%) | 1 693 (47.6%) | 405 356 (42%) | 8 416 (46.3%) |
| ACE inhibitor/ARB | 162 706 (74.3%) | 2 828 (79.5%) | 655 444 (68%) | 14 131 (77.8%) |
| Baseline cardiovascular disease was defined as ≥1 of hypertension, heart failure, ischemic heart disease, or arrythmia. Values are presented as n (%). Groups are stratified by cancer status and OHCA. ACE inhibitor/ARB, angiotensin-converting enzyme inhibitor/angiotensin receptor blockers; OHCA, out-of-hospital cardiac arrest. | | | | |

## Supplemental Figures

### Supplemental Figure 1

**Supplemental Figure 1:**

**Title: Flowchart of patient selection and included registers.**

**Caption**: The flowchart illustrates the selection of eligible cancer patients and matched controls, the exclusion process, and the Swedish national registers and corresponding years of data used to construct the study cohort.

### Supplemental Figure 2

**Supplemental Figure 2:**

**Title: Directed Acyclic Graph illustrating the causal model assumed for the regression modelling**

**Caption**: Directed Acyclic Graph including the exposure (green), outcome (blue with line), confounders (white), and assumed mediator (blue). CVD, cardiovascular disease; OHCA, out-of-hospital cardiac arrest.

### Supplemental Figure 3

**Supplemental Figure 3:**

**Title: Sensitivity Analysis: Adjusted OHCA Risk by Cancer Status and Site Including Same-Day OHCA and Cancer Diagnosis**

Caption: This figure presents hazard ratios (HRs) with 95% confidence intervals (CIs) for out-of-hospital cardiac arrest (OHCA) among individuals with cancer diagnosis compared with matched cancer-free controls, including cases in which OHCA and cancer diagnosis occurred on the same day. Estimates are presented overall (all cancers combined) and by cancer site. Associations were estimated using Cox proportional hazards models adjusted for age at cancer diagnosis or matching date, sex, Charlson Comorbidity Index, baseline cardiovascular disease, year of cancer diagnosis or matching, educational level, and income.

### Supplemental Figure 4

**Supplemental Figure 4:**

**Title: Adjusted OHCA risk by metastasis status**

**Caption:** This figure presents hazard ratios (HRs) with 95% confidence intervals (CIs) for out-of-hospital cardiac arrest (OHCA) among individuals with cancer diagnosis compared with matched cancer-free controls, stratified by metastasis status. Associations were estimated using Cox proportional hazards models adjusted for age at cancer diagnosis or matching date, sex, Charlson comorbidity index, baseline cardiovascular disease, year of cancer diagnosis or matching, educational level, and income.

### Supplemental Figure 5

**Supplemental Figure 5:**

**Title: Cumulative Incidence of OHCA in cancer patients and matched cancer-free controls over 10 years**

**Caption**: Unadjusted cumulative incidence of OHCA for cancer (red line) compared to matched cancer-free controls (blue line), for all cancers combined (any cancer) and by cancer site over a 10-year period. Second column presents subgroup analyses for females (dotted line) and males (solid line). Third column presents subgroup analyses for individuals <65 years (dotted line) and ≥65 years (solid line).

### Supplemental Figure 6

**Supplemental Figure 6:**

**Title: Adjusted OHCA risk by cardiovascular risk factor-related etiological group**

**Caption:** This figure presents hazard ratios (HRs) with 95% confidence intervals (CIs) for out-of-hospital cardiac arrest (OHCA) among individuals with cancer diagnosis compared with matched cancer-free controls, stratified by cardiovascular risk factor-related etiological cancer groups. The category *Any exposure-related cancers* includes all cancers assigned to at least one cardiovascular risk factor-related etiological group. Cancer sites associated with more than one cardiovascular risk factor were included in each relevant group. Associations were estimated using Cox proportional hazards models adjusted for age at cancer diagnosis or matching date, sex, Charlson comorbidity index, baseline cardiovascular disease, year of cancer diagnosis or matching, educational level, and income.
